# UDP-*N*-acetyl-α-D-galactosamine:polypeptide *N*-acetylgalactosaminyltransferase from the snail *Biomphalaria glabrata* – structural reflections

**DOI:** 10.1007/s10719-019-09886-y

**Published:** 2019-08-08

**Authors:** Aysegül Turupcu, Peter Poliak, Christian Margreitter, Chris Oostenbrink, Erika Staudacher

**Affiliations:** 1 Institute for Molecular Modeling and Simulation, Department of Material Sciences and Process Engineering, University of Natural Resources and Life Sciences, Vienna, Muthgasse 18, 1190 Vienna, Austria; 2 Department of Chemical Physics, Faculty of Chemical and Food Technology, Slovak University of Technology in Bratislava, Radlinského 9, SK-812 37 Bratislava, Slovakia; 3 Randall Centre for Cell & Molecular Biophysics, King’s College London, New Hunt’s House, Guy’s Campus, London, SE1 9RT UK; 4 Department of Chemistry, Glycobiology, University of Natural Resources and Life Sciences, Vienna, Muthgasse 18, 1190 Vienna, Austria

**Keywords:** ppGalNAcT, GalNAc-transferase, O-glycosylation, Snail, *Biomphalaria glabrata*, Homology modelling

## Abstract

UDP-GalNAc:polypeptide GalNAc transferase (ppGalNAcT; EC 2.4.1.41) is the initiating enzyme for mucin-type O-glycosylation in animals. Members of this highly conserved glycosyltransferase family catalyse a single glycosidic linkage. They transfer an *N*-acetylgalactosamine (GalNAc) residue from an activated donor (UDP-GalNAc) to a serine or threonine of an acceptor polypeptide chain. A ppGalNAcT from the freshwater snail *Biomphalaria glabrata* is the only characterised member of this enzyme family from mollusc origin. In this work, we interpret previously published experimental characterization of this enzyme in the context of in silico models of the enzyme and its acceptor substrates. A homology model of the mollusc ppGalNAcT is created and various substrate peptides are modelled into the active site. We hypothesize about possible molecular interpretations of the available experimental data and offer potential explanations for observed substrate and cofactor specificity. Here, we review and synthesise the current knowledge of Bge-ppGalNAcT, supported by a molecular interpretation of the available data.

## Introduction

UDP-GalNAc:polypeptide GalNAc transferases [EC 2.4.1.41]) (ppGalNAcT) is the initiating enzyme for mucin-type O-glycosylation. It transfers an *N*-acetylgalactosamine (GalNAc) residue from an activated donor (UDP-GalNAc) to a serine (Ser) or threonine (Thr) of an acceptor polypeptide chain. The so formed Tn-antigen structure is often further elongated and modified by other glycosyltransferases by adding galactoses, *N*-acetylglucosamines, *N*-acetylgalactosamines, fucoses, sialic acids and other sugars depending on the organism, tissue and developmental stage.

Like other types of glycosylation mucin-type O-glycosylation is involved in recognition events, signalling and modulation of protein processing [1]. While some ppGalNAcTs are redundant and widely distributed over tissues and time, others are spatially restricted and temporally regulated with unique biological functions in health, or have implications in disease [2, 3]. For example, specific ppGalNAcTs have been identified to cause deadhesion in Ebola infection or play an important role in the regulation of cholesterol transport [4, 5]. In humans 20 distinct genes are expressed encoding for 20 isoforms of ppGalNAcT which are well investigated regarding their structure, substrate specificities and function. In invertebrates homologous enzymes have been described in insects (*Drosophila melanogaster, Bombyx mori*), worms (*Caenorhabditis elegans, Fasciola hepática, Echinococcus granulosus*), parasites (*Toxoplasma gondi*, *Trypanosoma cruzi, Cryptosporidium* species) and the purple sea urchin (*Stronglylocetrotus purpuratus*) [6–16], but never in bacteria, plants or fungi. A ppGalNAcT from the freshwater snail *Biomphalaria glabrata* (GenBank: KC182513) is the only characterised member of this enzyme family from mollusc origin [17, 18].

The members of this enzyme family (CAZy^1^ glycosyltransferase family 27) are type II transmembrane proteins that share common structural features: a short N-terminal cytoplasmic tail, followed by the transmembrane region, a stem section, a first catalytic domain (subdomain A) containing a substrate and a manganese binding site, a second catalytic domain (subdomain B) containing the Gal/GalNAc motif responsible for binding of UDP-GalNAc, a flexible linker, and finally, a ricin-like lectin domain with a β-trefoil fold built from three repeat units at the C-terminus [2].

ppGalNAcTs appear structurally and functionally conserved in evolution. All of them display a GT-A fold but can be clustered in groups and subgroups by some differences in their primary structures [2]. The enzyme from *Biomphalaria glabrata* is a homologue of human ppGalNAcT2 and therefore belongs to group Ib [17].

Here we show the structural modelling of the mollusc ppGalNAcT from *Biomphalaria glabrata* (Bge-ppGalNAcT), comparing the snail enzyme and the well investigated homologous human ppGalNAcT2 with a focus on structural similarities and differences. We have derived an in silico model of the mollusc Bge-ppGalNAcT, and use this to shed light on new and previously obtained data from in vitro experiments. We consider this work as a review and synthesis of the current knowledge of Bge-ppGalNAcT, supported by a molecular interpretation of the available data.

## Material and methods

### Materials

*Spodoptera frugiperda* cells (Sf9, ATCC CRL-1711) were cultivated in IPL41 medium (SAFC Biosciences, St. Louis, USA) containing yeast extract, a lipid mixture supplemented with 10% fetal calf serum, at 27 °C [19]. Acceptor peptides were obtained from Cellmano Biotech Co., Ltd., Shanghai, China: Muc2 (PTTTPITTTTTVTPTPTPTGTQTK), CHT1 (APPAHPGPTPGPRPAPG), CHT2 (APPAHPGVTPGPRPAPG), CHT4 (APPAHPGPTPGKRPAPG), CHT5 (APPAHPGPTPGHRPAPG), CHT9 (APPAHPGPTEGPRPAPG), CHT11 (APPAHPGPTPRPRPAPG), CHT22 (APPAHPGPTPAPRPAPG).

### Expression of ppGalNAcT from *Biomphalaria glabrata*

A cDNA library was synthesized from embryonic cells from *Biomphalaria glabrata* (Bge cells, NR-40248, BEI Resources, NIAID, NIH). The enzyme, Bge-ppGalNAcT, without cytoplasmic tail and transmembrane domain (amino acid 1–26) was expressed and purified exactly as in [17]. A further truncated version, ΔppGalNAcT, omitting the complete lectin domain (lacking amino acid 477–600) was obtained in the same way. Protein concentrations were determined by the Micro-BCA protein assay (Pierce, Bonn, Germany) with bovine serum albumin as the standard.

### Determination of Bge-ppGalNAcT activity

The enzyme activity of Bge-ppGalNAcT and ΔppGalNAcT was determined in 20 μl reaction mixture containing 50 mM MES (2-(*N*-morpholino) ethanesulfonic acid), pH 7.0, 10 mM MnCl_2_, 40 nmol UDP-GalNAc (Sigma-Aldrich, Vienna, Austria), 20 μg acceptor peptide (Cellmano Biotech Co., Ltd., Shanghai, China) and 2 μl enzyme solution (ppGalNAcT or ΔppGalNAcT, protein concentrations 5.0 μg/ml) at 37 °C for 90 min. For evaluation of the influence of nucleotides the standard incubation assay was done in the presence of 0.05 mM. 0.1 mM, 0.2 mM, 0.4 mM or 0.75 mM UMP, UDP, UTP, ADP or GMP. For evaluation of the influence of monosaccharides the standard incubation assay was done in the presence of 0.2 mM. 0.5 mM, 1.0 mM, 2.0 mM or 3.75 mM glucose, galactose, N-acetylglucosamine or N-acetylgalactosamine. Each assay was carried out at least in duplicate and with appropriate controls.

Qualitative analysis was performed on an Autoflex Speed MALDI-TOF (Bruker Daltonics, Germany) mass spectrometer equipped with a 1000 Hz Smartbeam.II laser in positive mode using α-cyano-4-hydroxycinnamic acid as matrix (1% (*w*/*v*) in 65% (*v*/*v*) acetonitrile solution). For crystallization 1 μl of an 1:40 dilution of the samples was spotted on the plate, air dried, covered by 1 μl of matrix solution and again air dried. Spectra were processed with the manufacturer’s software (Bruker Flexanalysis 3.3.80).

Quantitative analysis was performed by HPLC on a reversed phase C18 column (4.6 × 250 mm, 5 μm, Thermo Scientific, Vienna, Austria) in 0.5% (*v*/*v*) trifluoroacetic acid in water, applying a linear gradient from 15 to 25% of eluent (0,1% (*v*/*v*) trifluoroacetic acid in acetonitrile) in 20 min at a flow rate of 1 ml/min with a detection at 220 nm. All quantitative values were calculated from the area of HPLC patterns.

### Molecular modelling

A homology model of Bge-ppGalNAcT from *Biomphalaria glabrata* was made using the SwissModel server [20–22]. We used two crystal structures of human ppGalNAcT2 (PDB ID 2FFV [23] and 4D0T [24]) as a template. These structures differ in the conformation of a flexible loop, which is open in the 2FFV structure and closed in the 4D0T structure.

The coordinates of UDP-GalNAc and Mn^2+^, were taken from the 4D0T crystal structure after aligning the catalytic domain of the modelled enzyme for both open and closed models. The molecular operating environment (MOE, Chemical Computing Group Inc.: Montreal, QC, Canada, https://www.chemcomp.com/, version 2018.0101) was used to minimize the potential energy to relieve steric clashes of the protein while keeping all the ligands and ions restrained. The Amber10 force field was used to describe molecular interactions [25]. The model peptide PGPTPGPR used in the experiments [18] was modelled by mutating the ligand TTPAPT which was aligned from the 4D0T crystal structure. When introducing the appropriate sidechains, the rotamers with the lowest potential energy were selected. A further minimization was performed to optimize the peptide coordinates, while keeping the enzyme restrained.

### Molecular dynamics simulations

Molecular dynamics simulations were performed for peptides Ac-PTP-NHMe, Ac-PSP-NHMe and Ac-VTV-NHMe for 100 ns each. Simulations were performed in explicit SPC water [26], at 300 K using the GROMOS11 simulation package and the GROMOS 54A7 force field [27, 28]. Conformational preferences of the backbone and the Thr / Ser side chains were monitored over the trajectories.

## Results and discussion

**Fig. 1:**
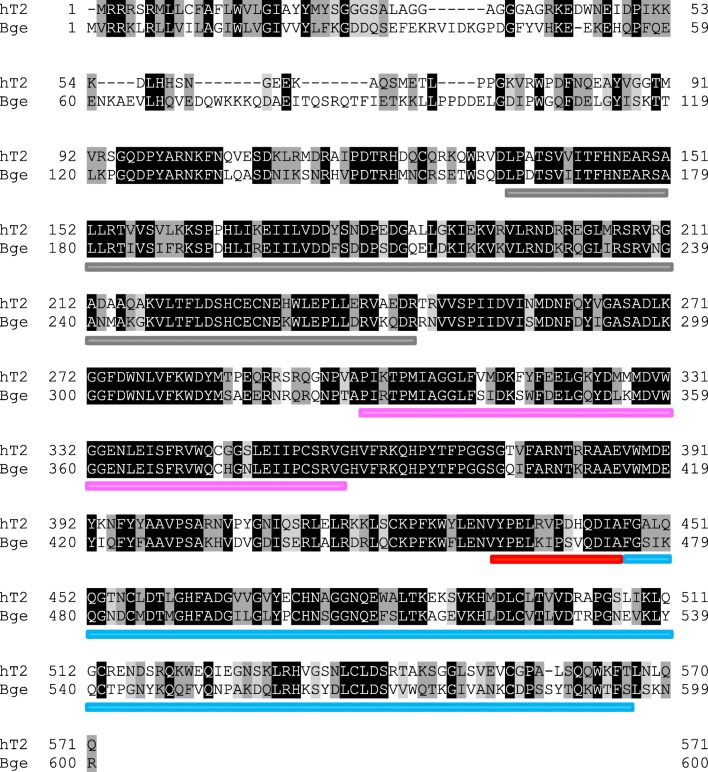
**Alignment of human ppGalNAcT2 (hT2, Q10471, GALT2_HUMAN) and modelled*Biomphalaria glabrata*ppGalNAcT (Bge, KC182513, S5S833_BIOGL)**. Homology of amino acids: identity - black background & white letter, high similarity: dark grey background & black letter, low similarity: light grey background & black letter, no similarity: white background & black letter. Coloured bars: catalytic subdomain A (grey), catalytic subdomain B (pink), linker loop (red), lectin domain (blue)

**Fig. 2:**
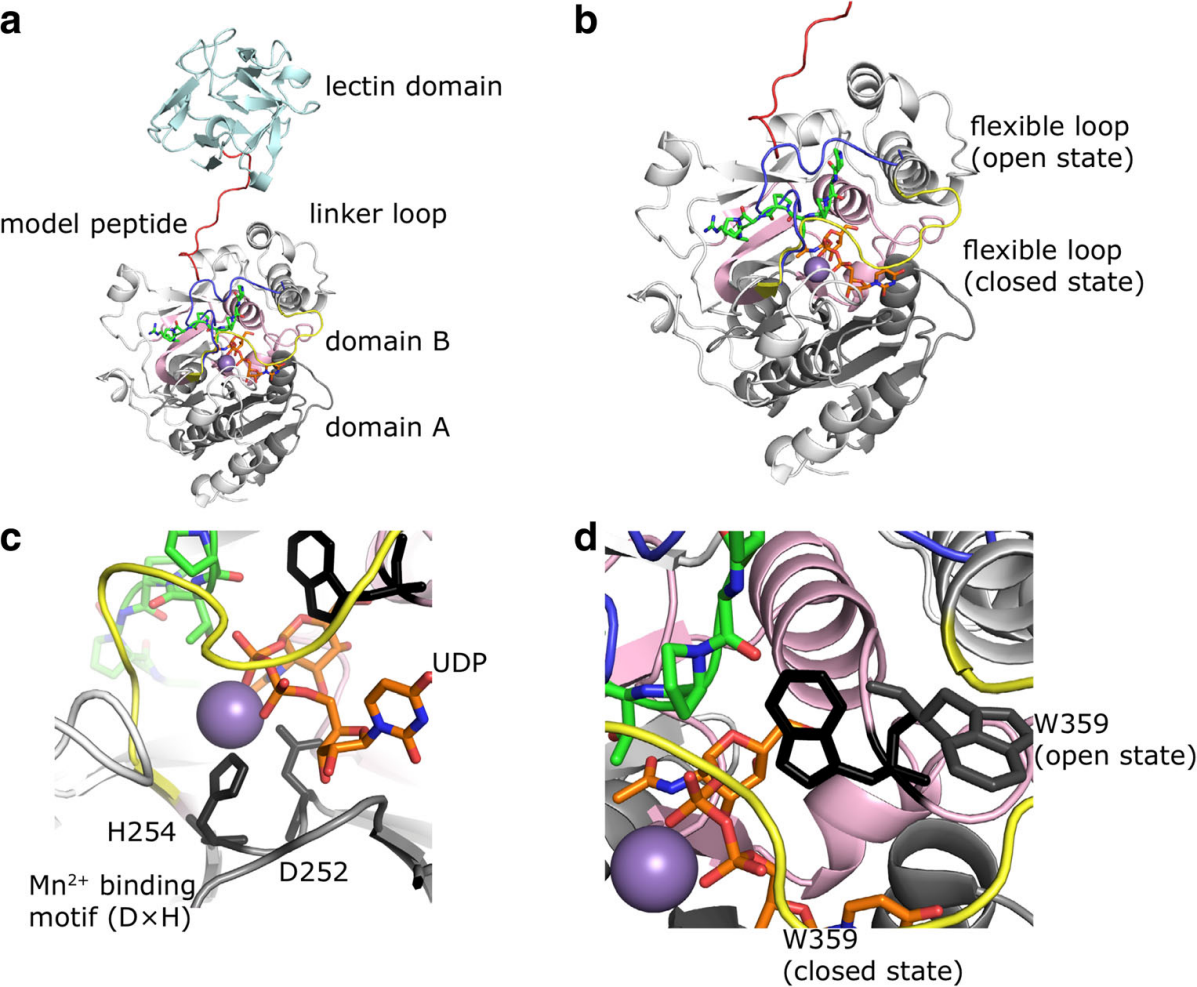
**Homology model of*Biomphalaria glabrata*ppGalNAcT**. **a** Overall structure of the modelled snail enzyme in complex with UDP (sticks in orange) and the glycopeptide PGPTPGPR (sticks in green). Enzyme in closed conformation shown in white; subdomain A and B shown in grey and pink; lectin domain shown in cyan with the connecting long linker (red) to the N-terminal domain. **b** The flexible loop is shown in blue for open conformation and in yellow for closed conformation which are modelled based on pdb entries 2FFV and 4D0T, respectively. **c** Active site with close-up view of the Mn^2+^-binding motif (DXH) where Mn^2+^ is shown as purple sphere and UDP in orange sticks. **d** Key catalytic residue W359 (W331 in human ppGalNAcT) is represented in black sticks with in/out conformation in the closed/open state where the enzyme is active/inactive, respectively

*Biomphalaria glabrata* ppGalNAcT is homologous to human ppGalNAcT2 with 61% sequence identity when the 4D0T structure is used as a template with 94% coverage; using 2FFV it is 65% sequence identity, with 82% coverage (Fig. 1). Therefore, we used the structures of the well investigated and crystallized human enzyme as templates for structural analysis. Homology models based on 4D0T and 2FFV have QMEAN4 scores of −1.83 and − 1.73, respectively. This indicates high model quality since the score provides an estimate of the degree of nativeness when it is close to zero. Figure 2a gives an overview of the different domains in the resulting structure.

### Cation requirement

Glycosyltransferases are known to require divalent cations for their activity. For Bge-ppGalNAcT we found no activity in the presence of the complexing agent EDTA and an order of increasing activity using Ca^2+^ < Mg^2+^ < Co^2+^ < Mn^2+^, while Cu^2+^ completely abolished the activity [17]. Trying to explain this order we found that the activity of Bge-ppGalNAcT in the presence of various metals notably follows the Irving-Williams series of relative stabilities of metal complexes. In this series, the stability generally increases independently of the ligand in the order of **Mg**^**2+**^ < **Mn**^**2+**^ < Fe^2+^ < **Co**^**2+**^ < **Ni**^**2+**^ < **Cu**^**2+**^ > Zn^2+^ [29]. Except for Mg^2+^, the Bge-ppGalNAcT activity decreases in the same order, that is **Mn**^**2+**^ > **Co**^**2+**^ > **Mg**^**2+**^ > Ca^2+^ > Ba^2+^ > **Ni**^**2+**^ > > **Cu**^**2+**^ showing that the stronger the metal binds to the active site of the ppGalNAcT, the lower is its activity. The strong interactions of Cu^2+^ might cause a strong binding in the active site, disabling UDP to leave after the reaction and resulting in an inhibition of the enzyme. A computational study of manganese-dependent glycosyltransferases has shown that the interaction energies of Mn^2+^ and Mg^2+^ with the active site motifs of glycosyltransferases are similar [30, 31]. However, Mn^2+^ is more prone to replacement of the first solvation shell by the active site ligands than Mg^2+^. The smaller ionic radius and lower polarizability of Mg^2+^ also creates higher tension on the protein thus weaken the complex. These findings are in accordance with the stability series. The complex with Mg^2+^ may simply be too weak to stabilize the interaction between ppGalNAcT and UDP sufficiently, explaining why Mg^2+^ shows less activity than Mn^2+^. However, studies on these aspects are still scarce and these questions need to be addressed by further calculations.

### Catalytic domains

Analogous to the other members of this glycosyltransferase family, our model of Bge-ppGalNAcT is composed of a catalytic subdomain A (GT1 motif) involved in substrate binding and manganese coordination, corresponding to residues Leu163-Arg273 and the catalytic subdomain B (GalNAc-T motif) involved in the catalytic reaction and UDP-GalNAc binding, corresponding to Pro328-Gly386 (Figs. 1 and 2a, b).

**Fig. 3:**
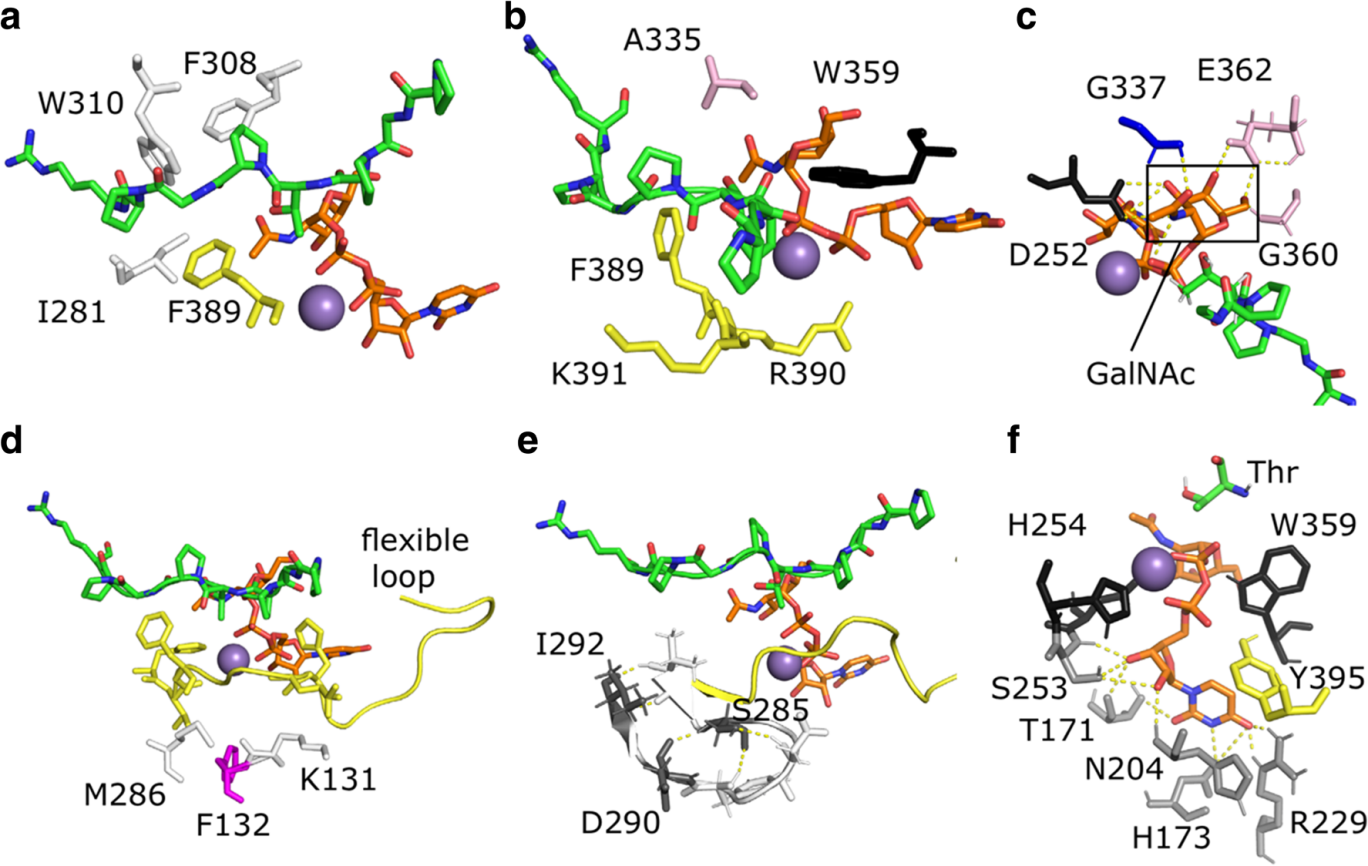
**Ligand interaction of the model with open loop and open lectin domain in complex with UDP-GalNAc (sticks in orange) and the ideal acceptor peptide Pro-Gly-Pro-Thr-Pro-Gly-Pro (sticks in green)**. Amino acids important for interaction are indicated (for numbering see Fig. 1). **a** Residues that are interacting directly with the peptide; Ile281, F308, Trp310 and F389 shown in sticks, colours are adapted from their domain classification (see Fig. 2) **b** Residues that are interacting directly with UDP indicating their importance in catalytic activity; Ala335, Trp359, Phe389, Arg390 and Lys391 shown in sticks. **c** Residues that are interacting with the N-acetyl moiety of GalNAc; Asp252 (black) and Gly337 (blue). Gly360 and Glu362 are interacting with HO4 and HO6 atoms of GalNAc respectively, depicted in sticks. **d** Phe132 residue (magenta) which has been found to inactivate the enzyme when mutated to Ser in human GalNAcT2. Met286 and Lys131 are suggested here to have similar effect through disrupting the dynamics of the flexible loop (yellow). **e** Residues (shown in grey) which are close to active site and differ from human ppGalNAcT2; Asp290 and Ser285. They might change the dynamics of the flexible loop resulting in a different arrangement upon substrate binding. **f** Polar interactions of uridine with the enzyme (yellow dashes) and the interacting residues: Thr171, His173, Asn204, Arg229 and Ser253 (shown in grey sticks similar to Fig. 2). Catalytic Thr (green sticks) and the important conserved residues that are interacting with the rest of the UDP moiety; His254 and Trp359 (black) and the residue from the flexible loop Tyr395 (yellow)

The enzyme contains the common DXH motif (Asp252-His254) and the highly conserved His387 for manganese binding (Fig. 2c). In the 2FFV crystal structure, the flexible loop is in an open conformation, turning away from the peptide binding cleft, while in 4D0T the loop is in a closed conformation, in contact with UDP. Lira-Navarrete et al. supplied further crystal structures of human ppGalNAcT2 with multiple conformations of this mobile loop in which 4D11 shows a semi-open conformation confirming the flexibility of this loop [24]. Dynamics of this loop is crucial for the interaction with the key catalytic residue Trp331 in human GalNAcT2 which corresponds to Trp359 in *Biomphalaria glabrata* (Fig. 2d). This Trp residue is referred to as “gate-keeper” in some publications [32]. When the enzyme is active, the loop is in the closed state and Trp359 adopts a conformation inside the active site. When the enzyme is inactive, the loop is in the open state and this residue is outside of the active site. The importance of the mobility of the loop was previously confirmed by mutagenesis studies with a Phe104Ser mutant, with the mutation located distantly from the active site but in crucial interaction with the flexible loop [33]. In Fig. 3 the most important amino acids involved in substrate and acceptor binding are shown. The model includes the substrate UDP-GalNAc and Pro-Gly-Pro-Thr-Pro-Gly-Pro as acceptor peptide in open loop and open lectin domain configuration (Fig. 3). The same highly conserved amino acids mediating acceptor peptide binding in approximately 20 ppGalNAcT isoforms are also present in Bge-ppGalNAcT. Residues interacting directly with the peptide: Ile281, Phe308 and Trp310 shown in Fig. 3a and residues interacting through the UDP moiety: Ala335, Trp359, Phe389, Arg390 and Lys391 shown in Fig. 3b. Furthermore, the effect of the important residue Phe132 (corresponding to Phe104 in the human form) on the dynamics of the flexible loop is illustrated in Fig. 3d. Additional residues Met286 and Lys131 are found to be interacting with the flexible loop which might be important for future experimental mutagenesis studies for Bge-ppGalNAcT.

The *N*-acetyl moiety of the UDP-GalNAc makes important interactions with the enzyme at Asp252 (Asp224 in human) and Gly337 (Gly309 in human) which may explain the observed restricted specificity of the Bge-ppGalNAcT for UDP-GalNAc versus UDP-Gal (see Fig. 3c). Although these residues are not unique for Bge-ppGalNAcT, they seem to have alternative roles in Bge-ppGalNAcT as compared to the human form. Asp252 is part of the DXH motif which mediates the Mn^2+^ in human, while in snail it is involved in interaction with UDP-GalNAc. It is striking to note that a shift has taken place with respect to the interactions with Gly. In human, Gly 308 (corresponding to Gly336 in Bge-ppGalNAcT) interacts with GalNAc, while this role is taken up by the neighbouring Gly337 in Bge-ppGalNAcT (corresponding to Gly309 in the human form). The slight differences in the arrangement of these residues in the active site, seem to be sufficient to explain the difference in specificity. Possibly, a tighter binding in the human form, e.g. by the deletion of Gly308, could lead to an increased specificity for GalNAc. In addition, the HO4 and HO6 groups of GalNAc are interacting with Glu362 and Gly360 as illustrated in Fig. 3c. The donor specificity seems to be important as human ppGalNAcT2 is suggested to be activated by a donor-induced mechanism [33]. Therefore, change in the donor from UDP-GalNAc to UDP-Gal might trigger re-arrangement of the active site through the flexible loop. The residues that differ from human ppGalNAcT2 mostly belong to surface residues (see Fig. 1 where the residues that differ from human ppGalNAcT2 are highlighted in grey). Among the distinct residues in Bge-ppGalNAcT, the loop connecting two beta-sheets changed from Asn257- Gln262 to Ser285-Asp290 which is nearby the flexible loop (see Fig. 3e). Grafting of the human loop into the snail enzyme, may further confirm, if these modifications are the cause of the observed structural rearrangements of the active site and differences in substrate specificity.

### Lectin domain

The ricin-like lectin domain of Bge-ppGalNAcT contains 121 residues at the C terminus (Phe475-Ser595) connected to the N-terminal domain with a long flexible linker (Val461-Ala474) (Figs. 1 and 2a). For human enzymes detailed studies have been carried out to determine the influence of the lectin domain on glycosylation behaviour. It was shown that in different isoforms the lectin domain is responsible for the direction of the acceptor peptide onto the catalytic domain in N- or C-terminal direction respectively [34, 35]. Due to the high flexibility of the linker between the catalytic part and the lectin domain it remains very challenging to capture any interaction between the lectin domain and the acceptor peptide in a reliable way. In in vitro experiments we have already shown, that the full enzyme does not display changed substrate specificity against small synthetic peptides compared to the ΔppGalNAcT (version expressed without lectin domain). Also with a larger peptide (Muc2) no differences were observed [18].

Many human ppGalNAcTs have one potential N-glycosylation site with the consensus structure Asn-X-Ser/Thr (for human ppGalNAcT2 this is Asn516-Asp517-Ser518) within their lectin-domain. However, there are no reports of an effective glycosylation. Bge-ppGalNAcT does not show any potential N-glycosylation site in its sequence.

### Inhibition experiments

**Fig. 4:**
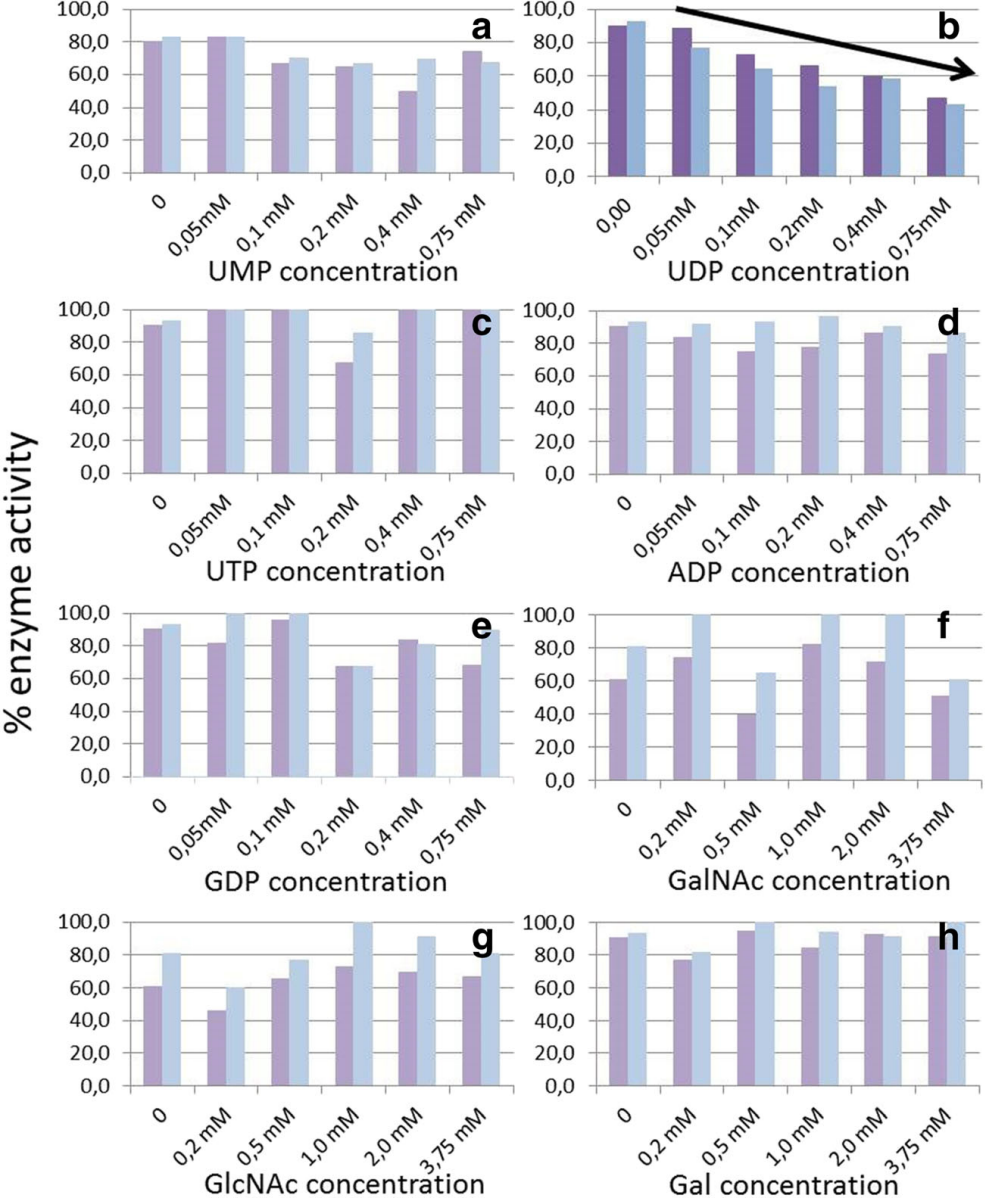
**Effect of increasing concentrations of nucleotides and sugars on Bge-ppGalNAcT activity**. **a** UMP; **b** UDP; **c** UTP; **d** ADP; **e** GDP; **f** GalNAc; **g** GlcNAc; **h** Gal. Left bars: enzyme expressed with lectin domain (purple), right bars: enzyme expressed without lectin domain (blue)

The nucleotides UMP, UDP, UTP, ADP and GDP were checked for their inhibition potential in the standard incubation assay (Fig. 4a–e). Only for UDP an inhibition effect was experimentally observed. Neither nucleotides with a different number of phosphate groups nor nucleotides with other bases than uridine inhibited the enzyme activity. No significant differences between the enzyme expressed with lectin domain and the version without lectin domain were determined. The exclusiveness for the nucleoside uridine was further investigated through a structural model where specific hydrogen bonding interactions were found between uridine and the enzyme. In addition to hydrogen bonds with residues Thr171, His173, Asn204, Arg229 and Ser253; conserved residues His254 and Trp359 of the enzyme and Tyr395 in the flexible loop region were found to be involved in uridine binding (Fig. 3f). Apparently, compounds with more or less phosphate groups (due to their size), or different nucleobases are not able to bind at this site. Furthermore, sugars (galactose, *N*-acetylglucosamine, *N*-acteylgalactosamine) did not show any inhibition of enzyme activity (Fig. 4f–h).

### Acceptor preferences

Analysis of native O-glycosylated proteins as well as incubation experiments with various human ppGalNAcTs revealed a general pattern for the minimal requirement for proper O-glycosylation sites [36–38]. They are mostly located in clusters of the peptide chain which contain mainly Pro, Ser and Thr and are sometimes referred to as Ser/Thr/Pro-rich domains. Near the O-glycosylation sites neither Trp, Leu, Ile and Phe are seen, nor other large amino acids, causing steric hindrance. Although there is no consensus sequence for O-glycosylation, importance of the Pro at certain positions (−3, −1, +1, +3) has been confirmed to be the most frequent residue near the modified Ser or Thr of mucins including its promoting effect on mucin-type O-glycosylation (“+” or “- “refers to residues that are N-terminal or C-terminal to a hydroxyl amino acid. The number reflects the distance from the hydroxyl amino acid). Isoenzymes of ppGalNAcTs which prefer to glycosylate acceptor substrates with a Thr/Ser-Pro-X-Pro sequence, where “X” may be any small hydrophobic amino acid, contain three highly conserved aromatic amino acids (Phe280, Trp282, Phe361 in human ppGalNAcT2) which interact with the acceptor [34, 38, 39]. The corresponding amino acids in the snail Bge-ppGalNAcT are Phe308, Trp310 and Phe389 (Fig. 3a).

In order to confirm our previous in vitro results regarding substrate preferences of Bge-ppGalNAcT, we modelled complexes of the enzyme with different substrate acceptors. In total seven peptides were selected, which are mostly covering the four groups we observed in [18] (Table 1). Group 1: very good acceptors with 95–100% incorporation of the GalNAc under standard conditions; Group 2: moderate acceptors with 50–94% incorporation; Group 3: weak acceptors with 5–49% incorporation; Group 4: incorporation less than 5%. Peptides with only one amino acid substitution were chosen to simplify the identification of potential causes. As described in the methods section, the sidechains of the ideal peptide were mutated and the sidechain rotamer with the lowest energy was selected. Next, a restrained energy minimization was applied only to the peptide using MOE and the Amber10 forcefield.

**Table 1:** Models were created from ideal peptide (PGPTPGPR) and open lectin with close loop conformation

Peptide		Incorporation of GalNAc in assay [%]	Change in position	Change
Name	Structure			
CHT1	PGP**T**PGPR	100	–	–
CHT9	PGP**TE**GPR	100	+1	Pro→Glu
CHT11	PGP**T**P**R**PR	100	+2	Gly → Arg
CHT2	PG**VT**PGPR	75	-1	Pro→Val
CHT5	PGP**T**P**H**PR	50	+2	Gly → His
CHT22	PGP**T**P**A**PR	50	+2	Gly → Ala
CHT4	PGP**T**PG**K**R	10	+3	Pro→Lys

**Fig. 5:**
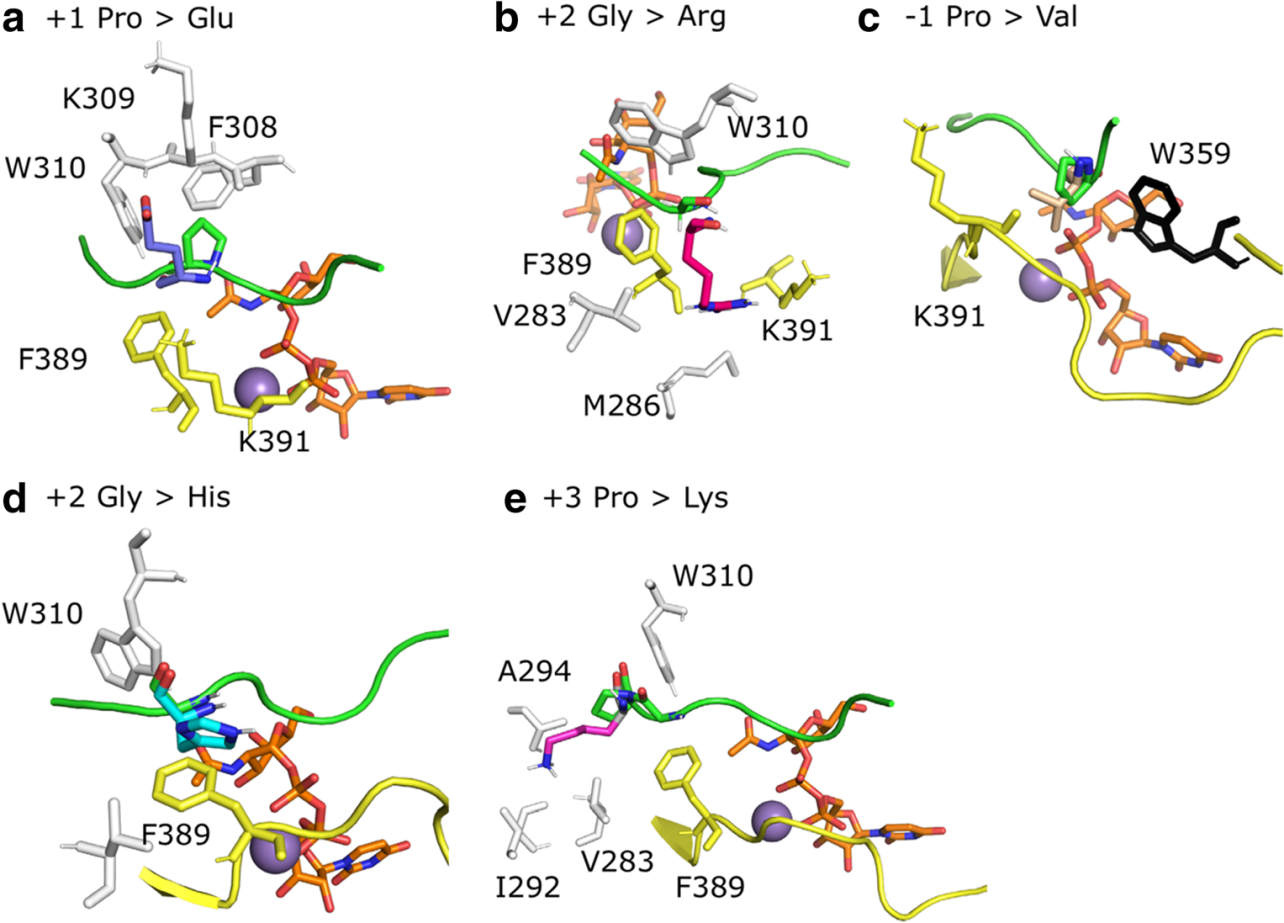
**Structural models with modified peptides**. Close up view of the modified peptides and the interacting residues of the enzyme are depicted in sticks on to the ideal peptide CHT1, PGPTPGPR shown in green, UDP-GalNAc in orange. Mn^2+^ shown as purple sphere. **a** Peptide CHT9 with change in position +1 growing Glu (in purple) showing 100% incorporation experimentally. **b** Peptide CHT11 (in position +2 growing Arg (in pink) showing 100% incorporation. **c** Peptide CHT2 (in position −1 growing Val (in wheat) showing 75% incorporation. **d** Peptide CHT5 (in position +2 growing His (in cyan) showing 50% incorporation. His instead of Gly creates an additional ring to the catalytic site environment; however, it is not disrupting the activity entirely. This might be due to π-π interactions with Phe389. **e** Peptide CHT4 (in position +3 growing Lys (magenta) showing 10% incorporation. Here, Lys is growing towards hydrophobic residues creating an unfavourable environment which might cause a rearrangement, resulting in low incorporation

Modelling of these peptides offers some rationalization of the experimentally determined incorporation results (Fig. 5). A change in +1 position to Glu (CHT9) or in +2 position to Arg (CHT11) showed a rather limited effect (Fig. 5a, b). Figure 5b shows how the newly introduced Arg at position +2 can be accommodated in a solvent-exposed way, without changing the conformation of the peptide. Even though the change introduced upon mutation to Val in −1 positon is small, its vicinity to the important catalytic residue Trp359 might cause the small amount of reduction in activity to 75% (Fig. 5c). Modification of the +2 position into His (CHT5) or Ala (CHT22), respectively, moderately changed the conformation of the peptide, potentially leading to a lower incorporation rate. In case of His, π-π interactions with Phe389 may be formed, possibly compensating for the unexpected occurrence of an aromatic side chain (Fig. 5d). In peptide CHT4, the optimal position of the newly introduced Lys in +3 position was found to be within a cluster of hydrophobic residues on the enzyme, which can be expected to be unfavourable for binding of the peptide (Fig. 5e). In vitro the activity is significantly reduced for this peptide.

Experimentally Bge-ppGalNAcT shows a clear preference for Thr acceptor sites over Ser acceptor sites. While for human enzymes a pocket fixing the methyl group of Thr has been proposed [23, 40], we suggest an alternative explanation due to the conformational preferences of the Thr and Ser sidechains. In 100-ns molecular dynamics simulation of Ac-PTP-NHMe and Ac-PSP-NHMe tripeptides, the conformational preferences of the χ_1_ dihedral angle were observed to be significantly different. For Thr, the conformation χ_1_ = +60° is observed for 70% of the time, while the conformations with χ_1_ = −60° and χ_1_ = 180° are both observed for 15%. For Ser, the χ_1_ = 180° conformation is observed for 55% of the time, and the χ_1_ = −60° and χ_1_ = +60° conformations are observed 25 and 20% of the time, respectively. For an optimal transfer of the GalNAc, the sidechain should be in a + 60**°** conformation, corresponding to the preferred conformation of the PTP tripeptide, but not of the PSP motif. The energetic and entropic penalty of bringing the PSP motif in an appropriate conformation offers an alternative explanation to the preference of ppGalNAcT for Thr acceptor sites.

### Order of multi-GalNAc incorporation

Bge-ppGalNAcT is able to glycosylate non-glycosylated as well as already glycosylated acceptor substrates. The choice of the first glycosylation site and the order of further ones follows exactly the above mentioned preferences. For human ppGalNAcT2 a glycosylated neighbouring Thr or Ser residue is nearly inhibitory, therefore its catalytic domain readily glycosylates non-glycosylated “naked” peptide substrates [34]. Also Bge-ppGalNAcT did not glycosylate a neighbour of an already glycosylated Thr. Muc2 was glycosylated by Bge-ppGalNAcT with 8 GalNAc-residues in total: PT^2^TT^4^PITT^8^TT^10^TVT^13^PT^15^PT^17^PT^19^GT^21^QTK (glycosylation is indicated by the given position number of Thr) [18]. Thr^19^ and Thr^21^ were found to be alternatives, we never found them glycosylated within the same peptide. Thr^15^ was the very first choice of the enzyme for glycosylation, which can be explained by its top ranked position with a Pro in position −1, +1 and + 3. On second rank we found T^2^ and T^13^ with Pro in +3 and − 1/+1 position, respectively. The −1 and + 3 positions for Pro have been predicted to be essential [36, 37], but the snail enzyme did not make any difference between −1 or + 1 Pro in combination with the +3 Pro, but perhaps a Val in the - 1 position is a similar enhancer like Pro [36]. All further glycosylation sites lacked a Pro in +3. In these cases the number of flanking Pro residues seemed to be the main regulation factor – with one exception: T^10^, with no Pro in neighbouring positions, was similarly ranked as T^17^, with Pro in −3, −1 and + 1 position. The only explanation for that is that the already glycosylated T^13^ in +3 position from T^10^ acts alike a Pro residue. All preferences are emphasizing the importance of the peptide backbone conformation. We have monitored the conformational preferences of Ac-VTV-NHMe and Ac-PTP-NHMe tri-peptides to see the effect of Pro on the backbone conformation. There was a significant increase in the polyproline II (P_II_) content from 37% to 83% with a decrease in the α content from 37% to 0% when Thr was in the Pro rich backbone. This result confirms that Pro changes the Thr preference towards more extended conformation, preparing the peptide for binding to the enzyme binding site cleft.

## Conclusion

In this study we have extended our earlier studies on Bge-ppGalNAcT by structural comparison with human ppGalNAcT2. Both are close relatives in the evolutionary highly conserved large family of ppGalNAcTs with 61% sequence identity with the human ppGalNAcT2 structure template used in this study. While the enzymes can be expected to be structurally similar, they show some differences in substrate and donor specificity.

We could confirm the importance of a number of highly conserved amino acids in the catalytic domains relevant for binding of the acceptor peptide as well as the substrate UDP-GalNAc. However, some small differences in the interaction with the *N*-acetyl moiety of the GalNAc residue can explain the fact, that Bge-ppGalNAcT is restricted to UDP-GalNAc as a donor [18], while human ppGalNAcT2 is active with UDP-GalNAc as well as UDP-Gal as donor substrates [41]. Furthermore we give an additional explanation for the preference of manganese as the divalent cation co-factor. The inhibition ability of UDP was shown by in vitro assays and confirmed by the model.

Regarding the acceptor peptide preferences we show structural models with various substrate peptides offering explanations for in vitro obtained activity data and suggest a new explanation for the Thr over Ser acceptor preference. In terms of multiglycosylation the snail enzyme seems to be more promiscuous than the human homologue. The human enzyme prefers non-glycosylated acceptor substrates and can perform long-range glycosylation, which means it needs a distance of at least 6 amino acids towards the next glycosylation site [34, 42], Bge-ppGalNAcT is able to glycosylate non-glycosylated and already glycosylated acceptor peptides. It does not glycosylate directly neighbouring Thr residues, but just one amino acid as a separation between two glycosylation sites is sufficient.

To date Bge-ppGalNAcT is the only investigated member of the ppGalNAcT family from mollusc origin. No others have been identified yet. One may speculate that molluscs express far less isoforms of this family than vertebrates and therefore the (few) member(s) must be more general in their acceptors preferences.

## Footnotes

1 http://www.cazy.org/
